# Class II HLA Genotype Association With First-Phase Insulin Response Is Explained by Islet Autoantibodies

**DOI:** 10.1210/jc.2017-02040

**Published:** 2017-12-28

**Authors:** Maarit K Koskinen, Johanna Lempainen, Eliisa Löyttyniemi, Olli Helminen, Anne Hekkala, Taina Härkönen, Minna Kiviniemi, Olli Simell, Mikael Knip, Jorma Ilonen, Jorma Toppari, Riitta Veijola

**Affiliations:** 1 Department of Pediatrics, University of Turku and Turku University Hospital, Turku, Finland; 2 Immunogenetics Laboratory, Institute of Biomedicine, University of Turku and Turku University Hospital, Turku, Finland; 3 Department of Biostatistics, University of Turku, Turku, Finland; 4 Department of Pediatrics, PEDEGO Research Unit, Medical Research Center, Oulu University Hospital and University of Oulu, Oulu, Finland; 5 Children’s Hospital, University of Helsinki and Helsinki University Hospital, Helsinki, Finland; 6 Research Programs Unit, Diabetes and Obesity, University of Helsinki, Helsinki, Finland; 7 Folkhälsan Research Center, Helsinki, Finland; 8 Tampere Center for Child Health Research, Tampere University Hospital, Tampere, Finland; 9 Institute of Biomedicine, Research Centre for Integrative Physiology and Pharmacology, University of Turku, Turku, Finland

## Abstract

**Context:**

A declining first-phase insulin response (FPIR) is characteristic of the disease process leading to clinical type 1 diabetes. It is not known whether reduced FPIR depends on class II human leukocyte antigen (HLA) genotype, islet autoimmunity, or both.

**Objective:**

To dissect the role of class II HLA DR-DQ genotypes and biochemical islet autoantibodies in the compromised FPIR.

**Design, Setting, Participants:**

A total of 438 children with defined HLA DR-DQ genotype in the prospective Finnish Type 1 Diabetes Prediction and Prevention Study were analyzed for FPIR in a total of 1149 intravenous glucose tolerance tests and were categorized by their HLA DR-DQ genotype and the number of biochemical islet autoantibodies at the time of the first FPIR. Age-adjusted hierarchical linear mixed models were used to analyze repeated measurements of FPIR.

**Main Outcome Measure:**

The associations between class II HLA DR-DQ genotype, islet autoantibody status, and FPIR.

**Results:**

A strong association between the degree of risk conferred by HLA DR-DQ genotype and positivity for islet autoantibodies existed (*P* < 0.0001). FPIR was inversely associated with the number of biochemical autoantibodies (*P* < 0.0001) irrespective of HLA DR-DQ risk group. FPIR decreased over time in children with multiple autoantibodies and increased in children with no biochemical autoantibodies (*P* < 0.0001 and *P* = 0.0013, respectively).

**Conclusions:**

The class II HLA DR-DQ genotype association with FPIR was secondary to the association between HLA and islet autoimmunity. Declining FPIR was associated with positivity for multiple islet autoantibodies irrespective of class II HLA DR-DQ genotype.

## Teaser

First-phase insulin response in intravenous glucose tolerance tests is compromised in children with multiple biochemical islet autoantibodies independently of HLA-conferred risk of type 1 diabetes.

Insulin is stored in its active form inside pancreatic *β*-cell secretory vesicles and is secreted into the circulation in response to increased plasma glucose levels and other stimuli, such as amino acids (1). The first-phase insulin response (FPIR) in an intravenous glucose tolerance test (IVGTT) represents the Ca^2+^ influx‒triggered steep secretory peak of insulin resulting from a small fraction of the granules that are immediately available for release in the *β*-cells (2). An acceleration in the decline of FPIR may occur during the last 2 years before diagnosis of type 1 diabetes (T1D) (3, 4). Indeed, we recently showed that FPIR is decreased as early as 6 years before the diagnosis of T1D (5).

Both genetic and environmental factors play a role in the pathogenesis of T1D (6, 7). The most important genetic determinants of T1D risk lie within the class II human leukocyte antigen (HLA) region, which appears to be involved in the initiation of islet autoimmunity as reflected in the appearance of islet autoantibodies, including insulin autoantibodies (IAAs), glutamic acid decarboxylase antibodies (GADAs), insulinoma-associated protein-2 antibodies (IA-2As), and zinc transporter-8 autoantibodies (ZnT8As) (8–10).

The aim of this study was to explore the role of HLA DR-DQ genotypes and islet autoantibodies in the compromised FPIR preceding T1D by analyzing the first available FPIR after seroconversion and changes in FPIR over time in children participating in regular follow-up in the prospective Finnish Type 1 Diabetes Prediction and Prevention (DIPP) study (11). Previous family studies have shown that autoantibody-positive siblings of children with newly diagnosed T1D carrying DQB1 risk genotypes had a lower FPIR than autoantibody-positive siblings with other genotypes (12). Furthermore, the FPIR was often reduced after the detection of autoantibodies in the DIPP study children (13). Thus, we hypothesized that both HLA DR-DQ risk genotypes and the presence of islet autoantibodies are associated with reduced FPIR but the HLA association is explained by its effect on the appearance of autoantibodies.

## Subjects and Methods

### Design of the study

The DIPP study is an ongoing population-based study that was launched in 1994 (11). Infants from the general population are screened for HLA DR-DQ‒associated genetic risk for T1D using cord blood. HLA genotyping procedure and eligibility criteria for DIPP follow-up have been described previously (9).

According to the current HLA screening criteria, approximately 10% of Finnish children fulfill the eligibility criteria for follow-up, and this screening strategy has a sensitivity of 60% for identifying children who will develop T1D. Eligible children were invited to the follow-up and regularly tested for signs of islet autoimmunity at 3- to 12-month intervals depending on age, autoantibody status, and study center (14). After the detection of any islet autoantibodies [IAAs, GADAs, IA-2As, and/or islet cell antibodies (ICAs)], the IVGTTs for this study were performed according to a standard protocol (13, 15). Briefly, the test was carried out after overnight fasting. An intravenous cannula was inserted into the dorsal hand vein or antecubital vein of the child after the use of local anesthesia with lidocaine-prilocaine cream. A glucose dose of 0.5 g/kg (maximum, 35 g) in a 20% solution was infused intravenously within 3 minutes ± 15 seconds. Blood samples were collected at 5 and 0 minutes before the start of the infusion and at 1, 3, 5, and 10 minutes after the end of the infusion.

### *β*-Cell function

The FPIR was calculated as the sum of insulin concentrations at 1 and 3 minutes during the IVGTT. Because several study subjects had undergone more than one IVGTT, we calculated *Δ*FPIR as an additional variable on the basis of the change in FPIR [*i.e.,* the last FPIR minus the first FPIR divided by the time between the samples (in years)].

### Study subjects

**Figure 1.**
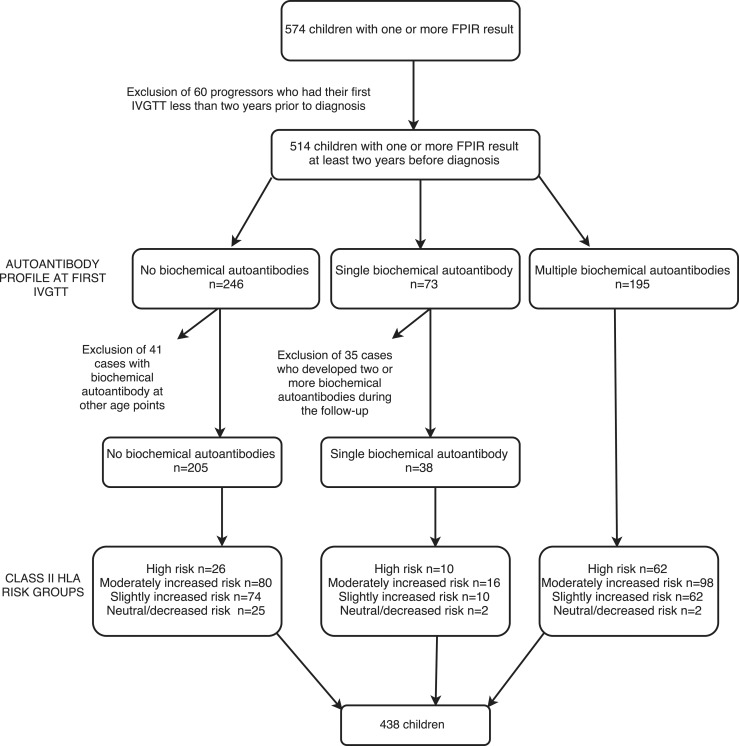
Flowchart of 574 children with HLA DR-DQ genotype who were observed from birth and underwent at least one IVGTT after the appearance of any islet autoantibody. The children were categorized according to the number of biochemical islet autoantibodies at the time of the first IVGTT.

The subjects included in this study are described in Fig. 1, which also shows their categorization into various autoantibody and HLA risk groups (described subsequently in detail).

A total of 574 HLA DR-DQ genotyped children observed from birth underwent at least one IVGTT after the appearance of any islet autoantibodies, including classic ICA (see flowchart in Fig. 1). Those subjects who had their first IVGTT performed during the last 2 years before diagnosis were excluded from the analysis to avoid the strong influence of a late preclinical decrease in FPIR. This resulted in the exclusion of 60 progressors from the initial data set because these children developed T1D <2 years after their first IVGTT.

Autoantibody status was defined on the basis of the presence of biochemical islet autoantibodies (IAAs, IA-2As, GADAs, ZnT8As) or ICAs only (later referred to as *no biochemical autoantibodies*) at the time of the first IVGTT. Data on ZnT8As were available from 509 of the subjects (99.0%).

A group of 246 children (47.9% of 514) had no biochemical autoantibodies at the time of the first IVGTT. Of these children, 205 remained persistently negative for biochemical autoantibodies.

Another group of 73 children (14.2% of 514) had a single biochemical autoantibody at the time of the first IVGTT, and 38 of these were positive for only a single biochemical autoantibody (referred to as *single biochemical autoantibody*), whereas 35 developed multiple (≥2) biochemical autoantibodies during the follow-up and were analyzed separately (Fig. 1; Supplemental Table 1).

A total of 195 children (37.9% of 514) had two or more biochemical autoantibodies (referred to as *multiple biochemical autoantibodies*) at the time of the first IVGTT and during the follow-up.

In the study cohort of 438 children who remained in the initial autoantibody group (268 males; 61.2%), the median age at the first IVGTT was 4.6 years (interquartile range, 2.7 to 7.3 years; range, 1.1 to 15.1 years). The total number of IVGTTs in these children was 1149. One hundred thirty-three children progressed to T1D during the follow-up, with a median age at diagnosis of 8.6 years (range, 3.8 to 18.7 years).

**Figure 2.**
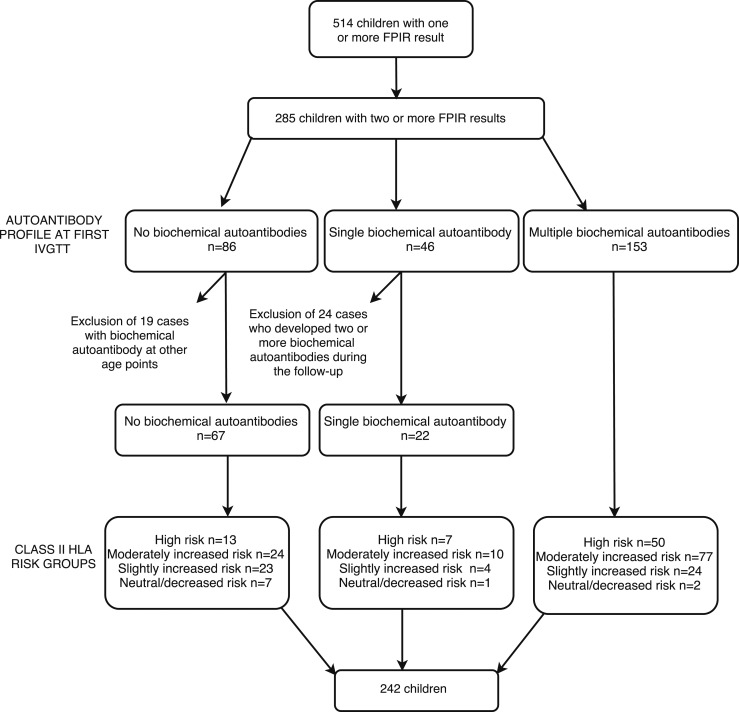
Flowchart of the 285 children with two or more FPIR results. The children were categorized according to the number of biochemical autoantibodies at the time of the first IVGTT.

Further analyses focusing on the longitudinal evolution of FPIR were performed in 242 children who remained in the initial autoantibody group (Fig. 2). The median interval between the first and the last IVGTT was 2.7 years (range, 0.4 to 14.5 years).

### HLA risk groups

The HLA DR-DQ genotypes of the children in the follow-up cohort were categorized into six risk groups: highly, moderately, or slightly increased risk; neutral risk; and slightly decreased or strongly decreased risk (Supplemental Table 2). This grouping is based on the presence of HLA DR-DQ haplotypes associated with T1D with variable strength: with increased disease risk, protection against it, or remaining neutral according to the data from the Finnish Pediatric Diabetes Register (9). The highly increased risk‒associated genotype combines risk-associated DR3 [(DR3)-DQA1*05-DQB1*02] and DR4 (DRB1*04:01/2/4/5-DQA1*03-DQB1*03:02) haplotypes, whereas the moderately increased risk means the combination of DRB1*04:01/2/5-DQA1*03-DQB1*03:02, the stronger risk haplotype, with a neutral haplotype. Further, the slightly increased risk means combinations of lower-risk haplotypes DRB1*04:04-DQA1*03-DQB1*03:02 or (DR3)-DQA1*05-DQB1*02 with a neutral haplotype or a combination of the strong-risk haplotype with a weakly protective haplotype. Neutral risk‒associated genotypes are various combinations of neutral haplotypes or combinations of a risk and a protective haplotype, and decreased risk‒associated genotypes are combinations of neutral and protective haplotypes or various protective haplotypes.

As the knowledge of HLA risk‒associated DR-DQ haplotypes and screening criteria evolved during the study period, some study subjects were actually found to carry neutral risk genotypes or, in a few cases, even decreased-risk genotypes despite initially being classified as carrying the increased risk‒associated DQB1 alleles. This allowed the inclusion of all these risk groups in the analyses. However, children with the most common strongly protective HLA-DQB1*06:02 allele were neither eligible nor enrolled in the DIPP follow-up. Because of the small number of children with neutral and decreased-risk genotypes, these were combined.

### Autoantibody analyses

IAAs, GADAs, and IA-2As were analyzed as described earlier (16). ZnT8As were measured by a radiobinding assay (17). Autoantibody status at the time of the first IVGTT was defined on the basis of autoantibody analyses performed within 60 days of the IVGTT. As a result of less frequent sampling, ZnT8As analyzed within 100 days from the first IVGTT were considered acceptable. One exception was made in the case of one child who gave several consecutive positive samples (the last one 1.5 years before the first IVGTT) and was considered to be positive for ZnT8A at the time of the first IVGTT, as it is highly unlikely that the child would have seroconverted back during that period (10).

### Statistical analyses

Because the response variable FPIR showed skewed distribution (assessed by the Shapiro-Wilks test and visual inspection), logarithmic transformation was applied to achieve the normality assumption in the statistical model. After the analyses, model-based means were back-transformed to the original scale while they represented model-based medians.

The relation between autoantibody status and HLA risk was analyzed with the *χ*^2^ test. Class II HLA DR-DQ risk was treated as a categorical variable from 1 to 4.

Cochrane-Armitage tests were applied to study the trends of two-categorical autoantibody groups in different HLA DR-DQ groups (no biochemical autoantibodies group vs multiple biochemical autoantibodies group; no biochemical autoantibodies group vs single biochemical autoantibody group; single biochemical autoantibody group vs multiple biochemical autoantibodies group).

Hierarchical linear models were used to analyze repeated measurements of FPIR. The number of IVGTTs decreased drastically after 5 years from the first test; thus, the period of 0 to 5 years from the first IVGTT was selected. The total number of IVGTTs in this period was 1023 (0 to 1 year with 523 values; 1 to 2 years with 191 values; 2 to 3 years with 149 values; 3 to 4 years with 93 values; and 4 to 5 years with 67 values; 89.0% of all). The maximum number of measurements per child was 10 (median, 2). Although the time intervals between the measurements varied between the subjects, time was modeled as a continuous factor in the statistical model. All available measurements at different time points were included. The statistical models included HLA and autoantibody status groups, as well as the interaction terms HLA × time and autoantibody group × time. The interaction term indicates that we studied whether the average change in FPIR over time was different between HLA groups and autoantibody groups. The model was also adjusted for age at the time of the first IVGTT. *P* values are shown for both an unadjusted model (for which only the autoantibody factor or HLA factor was included) and for an adjusted model (both HLA and autoantibody factors were included).

Statistical analyses were performed with JMP Pro version 11.2 and SAS^®^ System version 9.4 for Windows (SAS Institute Inc., Cary, NC). *P* < 0.05 (two-tailed) was considered statistically significant.

### Ethical considerations

The current study was conducted according to the guidelines of the Declaration of Helsinki and was approved by the local ethics committees in Turku, Oulu, and Tampere. Written informed consent was obtained from all subjects and/or their primary caretakers.

## Results

### Association between class II HLA risk group and autoantibody group

There was a strong association between HLA DR-DQ risk and autoantibody groups. Children in the increased class DR-DQ risk groups were more often positive for biochemical islet autoantibodies than those in other HLA risk groups (*P* < 0.0001 with the *χ*^2^ test) (Table 1). In a more detailed analysis, the difference was significant when children with no biochemical autoantibodies were compared with those with a single biochemical autoantibody or those with multiple biochemical autoantibodies (*P* < 0.02 and *P* < 0.0001, respectively, with the Cochrane-Armitage trend test). No positive trend was seen between the single autoantibody and multipositive group (*P* = 0.08).

**Table 1.** HLA-Conferred Risk for T1D Was Associated With the Number of Biochemical Islet Autoantibodies at the First IVGTT

Number of Biochemical Islet Autoantibodies	HLA Class II Conferred Susceptibility to T1D				
	High Risk, n (%)	Moderately Increased Risk, n (%)	Slightly Increased Risk, n (%)	Neutral/Decreased Risk, n (%)	Total
0	26 (26.5)	80 (41.2)	74 (63.2)	25 (86.2)	205
1	10 (10.2)	16 (8.3)	10 (8.6)	2 (6.9)	38
Multiple	62 (63.3)	98 (50.5)	33 (28.2)	2 (6.9)	195
Total n	98 (100)	194 (100)	117 (100)	29 (100)	423

*χ* ^2^ test; *P* < 0.0001. Number of children and proportions within various HLA risk groups are presented. In addition, pairwise comparisons were performed with the Cochrane-Armitage trend test: 0 vs 1 autoantibody, *P* = 0.02; 0 vs multiple autoantibodies, *P* < 0.0001; 1 vs multiple autoantibodies, *P* = 0.08.

### Analysis of FPIR using a hierarchical linear mixed model

Children within various HLA risk groups had significantly different FPIR median levels during follow-up (Table 2). Overall, HLA risk grading was inversely associated with FPIR. When using the hierarchical linear model, we first modeled the HLA risk alone and then analyzed the effect of time on FPIR. When the interaction between time and HLA group was included, FPIR over time was not significantly different between the HLA groups (*P* = 0.50).

**Table 2.** FPIR as Analyzed by Hierarchical Linear Mixed Model Including All Measured FPIR Values From the First IVGTT up to 5 Years of Follow-Up

	n*^a^*	FPIR Over Time, Median (IQR)	FPIR Over Time, Model-Based Median*^b^* (95% CI)	Unadjusted *P*	Adjusted *P**^b^*
HLA risk group					
High	275	46.6 (26.8, 79.1)	60.3 (53.5, 68.0)	0.008*^c^*	0.26*^b^*
Moderate	469	55.9 (38.9, 83.2)	69.6 (63.3, 76.5)		
Slightly increased	228	66.5 (45.2, 100.1)	64.6 (57.4, 72.8)		
Decreased/neutral	51	76.0 (58.4, 109.2)	70.5 (56.1, 88.6)		
Autoantibody group (number of biochemical autoantibodies)					
0	319	77.2 (58.4, 114.3)	83.1 (75.6, 91.4)	<0.0001*^d^*	<0.0001*^b^**^,^**^e^*
1	77	76.7 (57.4, 120.9)	75.4 (62.4, 91.1)		
Multiple	627	46.5 (29.7, 69.3)	46.1 (41.9, 50.8)		

Abbreviations: CI, confidence interval; IQR, interquartile range.

^a^ n = number of FPIR results.

^b^ Model-based medians and adjusted *P* values are from the model that included both HLA and autoantibody groups and their interaction with time.

^c^ This unadjusted *P* value represents the model including the HLA group, follow-up time (0‒5 years), and whether the median FPIR over time was different between HLA groups.

^d^ This unadjusted *P* value represents the model including the autoantibody group, follow-up time (0‒5 years), and whether the median FPIR over time was different between autoantibody groups.

^e^ Pairwise comparisons between groups with 0, one, or multiple biochemical autoantibodies (0 vs 1: *P* = 0.3; 0 vs multiple: *P* < 0.0001; 1 vs multiple: *P* < 0.0001).

**Figure 3.**
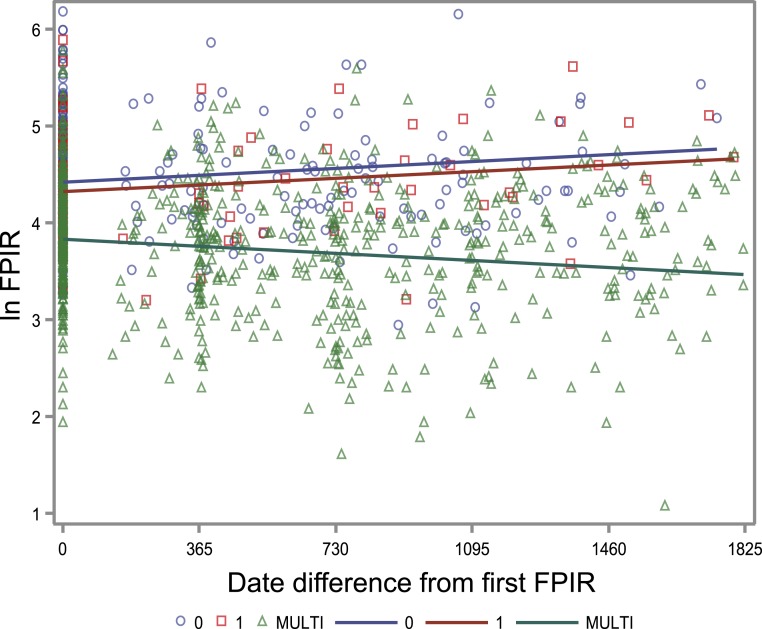
FPIR over time in children with various numbers of biochemical islet autoantibodies (0, single, or multiple). The *y*-axis shows the FPIR on a logarithmic scale. FPIR results from a total of 1023 IVGTTs are included. The *x*-axis shows time, and 0 indicates the time of the first FPIR. We used a hierarchical linear model to analyze repeated measurements of FPIR and to report coefficient estimates from the model, including both HLA group and autoantibody group and their interaction with time. The coefficient estimate was 0.000195, standard error 0.000060, *P* = 0.0013 for the group with no autoantibodies (blue circles). The corresponding values were 0.000188, standard error 0.000101, *P* = 0.063 for the group with a single autoantibody (red open squares), and −0.00020, standard error 0.000046, *P* < 0.0001 for the group with multiple autoantibodies (green open triangles).

When the autoantibody group was added to the model, HLA risk grading was no longer significantly associated with FPIR (*P* = 0.26), and FPIR change over time was not significant between HLA groups (*P* = 0.35). In contrast, positivity for multiple autoantibodies was strongly associated with lower FPIR both in unadjusted and HLA-adjusted models (*P* < 0.0001 for both) (Table 2). Furthermore, the change in FPIR over time was significantly different between multipositive children and those with single or no autoantibodies both in HLA-adjusted and HLA-unadjusted models (*P* < 0.0001 for both). FPIR decreased over time in children with multiple autoantibodies but increased slightly in others (Fig. 3).

### Further details of the change in FPIR over time

The longitudinal evolution of FPIR over time was determined as the change between the last and the first FPIRs per time in years (*Δ*FPIR) in children who participated in more than one IVGTT. The median values of the actual change in FPIR, as well as *Δ*FPIR in different HLA and autoantibody groups, are shown in Table 3. A significant decrease in FPIR was observed in all groups of multipositive children regardless of HLA-associated risk.

**Table 3.** Median of the FPIR at Baseline and Change in FPIR and *Δ*FPIR (Difference Between the Last and the Baseline FPIRs per Time in Years) According to Different Autoantibody Groups Within the HLA Risk Groups

Autoantibody Group^*a*^ Status Within the HLA Risk Group	Baseline FPIR, mU/L Median (95% CI)	Age at First IVGTT, y Median (IQR)	Change in FPIR, mU/L Median (95% CI) *(n*^*b*^*)*	Time Between Last and First IVGTT, y Median (range)	*Δ*FPIR, mU/L/y Median (95% CI)	Number of Progressors (%)
High (n = 98)	54.3 (46.6, 64.4)	3.5 (2.4, 6.6)	−5.2 (−9.0, −0.7) *(70)*	2.6 (0.6–11.3)	−1.9 (−4.1, −0.2)	49 (50)
0 (n = 26)	81.9 (63.3, 102.0)	4.9 (3.3, 7.9)	9.7 (−18.6, 66.3) *(13)*	2.6 (1.0–6.3)	2.7 (−7.3, 26.0)	0
1 (n =10)	64.8 (47.5, 128.6)	7.4 (6.1, 9.0)	11.0 (−90.3, 75.6) *(7)*	1.4 (0.6–7.4)	4.4 (−12.3, 54.9)	1 (10)
Multiple (n = 62)	41.3 (36.5, 49.5)	2.6 (2.2, 4.4)	−8.2 (−12.4, −2.9) *(50)*	2.8 (1.0–11.3)	−3.4 (−5.0, −1.0)	48 (77)
Moderate (n = 194)	67.0 (61.2, 72.5)	4.5 (2.7, 7.3)	−3.0 (−6.1, 2.0) *(111)*	2.8 (0.4–14.5)	−0.6 (−2.4, 0.7)	61 (31)
0 (n = 80)	75.3 (68.4, 91.9)	5.8 (3.2, 7.7)	21.3 (11.2, 35.8) *(24)*	2.1 (0.8–7.7)	8.8 (3.9, 16.0)	0
1 (n = 16)	84.9 (55.9, 120.9)	4.5 (2.5, 8.9)	−6.1 (−31.2, 81.2) *(10)*	2.7 (0.4–6.4)	−5.0 (−12.4, 12.6)	1 (6)
Multiple (n = 98)	53.7 (46.5, 63.3)	3.5 (2.3, 5.5)	−5.8 (−13.2, −2.0) *(77)*	3.1 (0.8–14.5)	−1.7 (−4.9, −0.5)	60 (61)
Slightly increased (n = 117)	69.3 (62.8, 80.2)	5.7 (3.7, 7.8)	−1.9 (−11.6, 7.8) *(51)*	2.8 (0.8–11.0)	−1.0 (−4.4, 3.1)	22 (19)
0 (n = 74)	80.4 (69.3, 97.0)	6.3 (4.5, 8.0)	7.8 (−1.0, 31.9) *(23)*	2.1 (0.8–11.0)	3.1 (−1.0, 12.9)	0
1 (n = 10)	65.7 (44.9, 150.0)	6.8 (3.6, 8.4)	45.8 (−5.7, 109.2) *(4)*	3.9 (3.1–5.9)	7.6 (−1.9, 26.3)	1 (10)
Multiple (n = 33)	51.3 (42.6, 60.2)	4.0 (2.8, 5.5)	−22.0 (−31.9, −7.5) *(24)*	3.1 (1.0–8.0)	−6.2 (−10.9, −1.9)	21 (64)
Neutral/decreased (n = 29)	71.8 (61.4, 110.9)	4.6 (3.9, 7.7)	9.0 (−33.6, 67.1) *(10)*	2.5 (0.9–7.4)	4.9 (−15.3, 37.5)	1 (3)
0 (n = 25)	69.4 (59.2, 110.9)	4.6 (3.8, 8.0)	33.2 (−33.6, 307.2) *(7)*	2.9 (0.9–7.4)	15.8 (−15.3, 41.3)	0
1 (n = 2)	98.7 (76.0, 121.5)	5.6 (4.5, 6.7)	3.2 *(1)*	2.1	1.6	0
Multiple (n = 2)	62.5 (53.1, 71.8)	4.5 (2.3, 6.8)	−30.3 (−37.8, −22.8) *(2)*	4.0 (2.0–6.1)	−11.3 (−18.9, −3.7)	1 (50)

Abbreviations: CI, confidence interval; IQR, interquartile range.

^a^ Number of biochemical autoantibodies.

^b^ Number of subjects.

The median of *Δ*FPIR was positive in the 19 children who had no biochemical autoantibodies at baseline but who developed more autoantibodies during follow-up. On the other hand, *Δ*FPIR tended to be negative in 24 children who had a single biochemical autoantibody at baseline and who developed more autoantibodies over time (Supplemental Table 1).

## Discussion

We show here that the presence of multiple autoantibodies inversely correlated with FPIR. The apparent HLA correlation with FPIR was secondary and was explained by HLA DR-DQ association with islet autoimmunity. In the unadjusted hierarchical linear model, the HLA DR-DQ risk group was significantly associated with FPIR; however, when autoantibody status was added to the model, the significance of the HLA DR-DQ was lost and the autoantibody association with FPIR remained highly significant. Overall, the HLA DR-DQ predisposition determines the risk of developing islet autoantibodies but does not affect the progression from autoantibody positivity to clinical T1D, which is in line with previous studies (9, 18).

The observation that FPIR declined during follow-up in children with multiple biochemical islet autoantibodies whereas FPIR slightly increased over time in the groups with single or no biochemical autoantibodies is an interesting finding. This observation suggests that at the first signs of humoral autoimmunity development, as indicated by the appearance of biochemical islet autoantibodies, *β*-cell function was not significantly changed compared with the group with no biochemical autoantibodies. Multiple autoantibodies may be associated with the extent of damage of the *β*-cell, which is reflected in the FPIR (19). Therefore, the FPIR was increasing in the group of children with only one autoantibody, as in the control group, whereas it was declining in children with multiple autoantibodies. Interestingly, subjects with multiple autoantibodies almost invariably develop T1D over time, whereas the progression rate to clinical T1D is much lower in those with a single autoantibody (16, 19–21).

Still, the mechanism by which changes observed in FPIR are linked to autoantibodies is unknown. *β*-cell autoimmunity may be secondary to the initial *β*-cell insult (8). This could occur during early development of the pancreas (22), when *β*-cell growth or differentiation may be compromised by a series of environmental factors, such as viruses or *β*-cell toxic compounds. The effect of HLA DR-DQ risk genotypes may be related to the order in which the autoantibodies appear during the disease process (14, 23, 24). Some as yet unknown environmental and genetic factors may contribute to the deterioration of FPIR and to a change from single autoantibody positivity to multipositivity. In addition, reversal from multiple autoantibodies to single or no autoantibodies is much less frequent than reversal from single to no autoantibodies (25).

The relationship between the longitudinal evolution of FPIR and the number of islet autoantibodies has not been investigated in detail. However, our findings are in line with those of Robert *et al.* (26), who noted that increasing FPIR was generally associated with decreasing ICA titers, whereas a stable or declining FPIR was associated with increasing ICA titers, which may reflect the presence of multiple biochemical islet autoantibodies (16).

In HLA-identical siblings of patients with T1D, the FPIR was lower than in haplo- or nonidentical siblings, and the increase in FPIR was also more modest (12). These children also presented with higher titers of GADA and ICA. Fasting blood glucose levels were similar between the three groups; however, during the IVGTT, the glucose disappearance rate (K_g_) was lower in HLA-identical children, reflecting a change in glucose homeostasis in these children.

In autoantibody-positive Finnish children under the age of 5 years who had an FPIR that was lower than that of the age-related reference, an increase in the second FPIR was seen in only one of the four children, whereas an increase in FPIR occurred in four of five children whose initial FPIR was above the normal range (13). This could highlight the importance of early *β*-cell growth. Interestingly, in a multinational study of ICA-positive first-degree relatives of patients with T1D, the FPIR was below the age-specific 10th percentile in 39% of participants who were below the median age of the study subjects (15.9 years), and impaired glucose tolerance was observed in the baseline oral glucose tolerance test in 12% of subjects, whereas the corresponding figures were 34% and 7% in older participants (23). The differences between the two age groups did not reach statistical significance, and it is noteworthy that only 3% of study subjects were younger than 5 years (23).

The long follow-up time in the DIPP study allowed us to investigate long-term changes in FPIR, which was possible in only a few earlier studies. Furthermore, to our knowledge, this is the largest study population of young children with FPIR data collected over a long-term follow-up. The youngest children in our series were <2 years of age. Many of the studies addressing normal values of FPIR have lacked data from the first years of life. Because islet autoantibodies have been continuously monitored in the DIPP study, we were able to perform detailed analyses of the associations between the number of biochemical islet autoantibodies and the evolution of FPIR. However, data about the pattern of FPIR in children who remain autoantibody negative is scarce, especially during the first years of life. In our series, children with only classic ICAs were considered islet autoantibody negative because the long-term experience from the DIPP study and other series has shown that the disease risk conferred by ICA alone is very low (16, 27).

Our results were derived mainly from young, prepubertal children (median age at first IVGTT, 4.6 years) and therefore may not be generalizable to older populations. The categorization of genotypes to specific risk groups can mean that the effect of individual genotypes is missed; for example, there can be interactions with other loci that change the overall risk conferred by genotype. The protective genotypes are common in the general population, and most of the Finnish population (76.9%) carries HLA DR-DQ genotypes associated with neutral or decreased risk for T1D. In this study, very few subjects had these genotypes because of the genetic screening performed from cord blood in the DIPP study (9). Our results show, however, that declining FPIR associated with multiple autoantibodies also occurred in children with a decreased class II HLA‒conferred risk for T1D.

To conclude, this study showed that the number of biochemical islet autoantibodies had a critical impact on the long-term evolution of FPIR, and the inverse correlation between the degree of class II HLA DR-DQ risk and FPIR was secondary to the association between the risk genotypes and the appearance of islet autoantibodies. The declining FPIR was associated with multiple autoantibodies irrespective of class II HLA DR-DQ risk group. An increase in FPIR over time was observed in children with no biochemical islet autoantibodies.

## Supplementary Material

Supplemental Table 1

Supplemental Table 2

## Abbreviations:

DIPP: Finnish Type 1 Diabetes Prediction and Prevention
FPIR: first-phase insulin response
GADA: glutamic acid decarboxylase antibody
HLA: human leukocyte antigen
IA-2A: insulinoma-associated protein-2 antibody
IAA: insulin autoantibody
ICA: islet cell antibody
IVGTT: intravenous glucose tolerance test
T1D: type 1 diabetes
ZnT8A: zinc transporter-8 autoantibody.
