# Supplementary material for: Class II HLA Genotype Association With First-Phase Insulin Response Is Explained by Islet Autoantibodies
Source: J Clin Endocrinol Metab. 2017 Dec 28;103(8):2870–8. doi: 10.1210/jc.2017-02040 (PMC6097602; doi:10.1210/jc.2017-02040)
Supplement: Supplemental Table 1 [file jc.2017-02040.st1.docx]

**Supplementary Table 1** The median change of first phase insulin response (FPIR) and the median of ΔFPIR (the difference between the last and the baseline FPIR per time in years) according to the autoantibody groups defined by the number of biochemical islet autoantibodies at baseline and during follow-up

| (*n*)^a^ | | | The baseline FPIR;mU/L  Median (95 % CI) | Age at the first IVGTT; years  Median (IQR) | Change in FPIR; mU/L  Median (95 % CI) *(n)*^a^ | Time between the last and the first IVGTT; years  Median (range) | ΔFPIR; mU/L/year  Median (95% CI) | Number of progressors  (%) |
| --- | --- | --- | --- | --- | --- | --- | --- | --- |
|  | | |  |  |  |  |  |  |
| **No bc aab^†^ at baseline but more autoantibodies during follow-up (41)** | | | 61.6 (52.0, 99.0) | 4.3 (3.4, 5.9) | 17.8 (8.6, 36.9) *(19)* | 2.1 (0.7–9.2) | 6.0 (3.4, 25.6) | 5 (12) |
| High risk (10) | | | 82.7 (51.5, 167.4) | 4.4 (3.0, 6.3) | 16.6 (-4.0, 107.7) *(6)* | 1.7 (0.7–9.2) | 12.8 (-3.8, 54.2) | 1 (10) |
| Moderately increased risk (22) | | | 60.8 (47.5, 106.6) | 4.0 (2.6, 5.7) | 25.5 (6.9, 146.8) *(8)* | 4.5 (1.9–6.0) | 4.9 (3.4, 31.8) | 4 (18) |
| Slightly increased risk (8) | | | 53.6 (31.0, 206.9) | 5.0 (4.1, 5.7) | 8.6 (-7.7, 95.0) *(5)* | 2.1 (0.9–3.0) | 3.8 (-8.2, 79.4) | 0 |
| Neutral/decreased risk (1) | | | 158.2 | 9.7 | *(0)* | *-* | *-* | 0 |
|  | | |  |  |  |  |  |  |
|  | **No bc aab at baseline and one autoantibody during follow-up (35)** | | 60.0 (49.0, 97.0) | 4.5 (4.0, 5.6) | 17.8 (8.2, 80.2) *(15)* | 2.0 (0.7–9.2) | 10.0 (1.7, 31.8) | 1 (3) |
|  | No bc aab and appearance of one autoantibody after the first IVGTT (14) | | 64.3 (42.6, 147.0) | 4.1 (2.6, 4.9) | 10.4 (-4.0, 32.7) *(9)* | 2.1 (0.9–9.2) | 3.8 (-3.8, 19.6) | 0 |
|  | No bc aab and appearance of one autoantibody before the first IVGTT (21) | | 60.0 (48.0, 99.0) | 5.3 (3.9, 6.5) | 55.9 (16.3, 107.7) *(6)* | 2.0 (0.7–4.4) | 29.6 (3.7, 79.4) | 1 (5) |
|  | | |  |  |  |  |  |  |
|  | **No bc aab at baseline and ≥2 autoantibodies during follow-up (6)** | | 83.3 (31.0, 169.3) | 3.4 (2.3, 5.6) | 14.6 (6.9, 36.9) *(4)* | 4.3 (2.0–6.0) | 3.6 (2.8, 6.2) | 4 (67) |
|  | | |  |  |  |  |  |  |
| **One bc aab at baseline but more autoantibodies during follow-up (35)** | | | 69.3 (49.0, 97.4) | 5.1 (3.5, 7.0) | -0.9 (-12.3, 18.3) *(24)* | 3.8 (1.5–9.1) | -0.3 (-4.6, 5.9) | 12 (34) |
| High risk (9) | | | 39.6 (69.6, 85.7) | 6.1 (3.1, 6.6) | -2.9 (-38.9, 19.4) *(7)* | 4.1 (3.1–7.6) | -0.8 (-12.2, 9.2) | 6 (67) |
| Moderately increased risk (22) | | | 65.5 (49.0, 145.5) | 4.4 (3.5, 7.5) | 1.0 (-25.6, 41.2) *(15)* | 3.4 (2.1–6.8) | 0.2 (-6.0, 7.0) | 6 (27) |
| Slightly increased risk (3) | | | 81.2 (56.1, 109.9) | 5.0 (4.7, 5.7) | -56.2 *(1)* | 3.9 | -14.3 | 0 |
| Neutral/decreased risk (1) | | | 111.3 | 11.2 | 147.0 *(1)* | 2.4 | 61.3 | 0 |
|  | |  |  |  |  |  |  |  |
|  | One bc aab at the baseline and the second bc aab after the first IVGTT (23) | | 85.0 (52.1, 110.2) | 5.2 (3.8, 7.3) | -2.9 (-37.7, 41.2) *(17)* | 3.7 (1.5–9.1) | -0.8 (-6.0, 9.2) | 6 (26) |
|  | One bc aab at the baseline and the second bc aab before the first IVGTT (12) | | 50.3 (32.6, 89.8) | 4.8 (3.5, 6.7) | 1.8 (-25.6, 19.4) *(7)* | 4.0 (2.1–7.6) | 0.5 (-12.0, 5.9) | 6 (50) |

**Notes:** Within each autoantibody group data are also given in various HLA risk groups. The data for children who had no biochemical autoantibodies at baseline but became autoantibody positive during follow-up (n=41) are presented in two further groups: the children who developed only one autoantibody (n=35) and those who developed two or more biochemical autoantibodies (n=6). The data for children with one autoantibody at baseline (n=35) are also presented in two further groups: the children with the second biochemical autoantibody after the first IVGTT (n=23) and those with the second biochemical autoantibody before the first IVGTT (n=12).

**Abbreviations:** bc aab, biochemical islet autoantibodies

^a^ number of subjects
