# Supplementary material for: Class II HLA Genotype Association With First-Phase Insulin Response Is Explained by Islet Autoantibodies
Source: J Clin Endocrinol Metab. 2017 Dec 28;103(8):2870–8. doi: 10.1210/jc.2017-02040 (PMC6097602; doi:10.1210/jc.2017-02040)
Supplement: Supplemental Table 2 [file jc.2017-02040.st2.docx]

**Supplementary table 2** HLA class II genotype grouping according to the risk for type 1 diabetes

| **Risk** | ***n (% of total n=438)*** | **HLA genotype description** |
| --- | --- | --- |
| High risk | 98 *(22.4)* | heterozygosity for the two different risk-associated haplotypes:  DRB1*04:01/2/4/5-DQA1*03-DQB1*03:02 / (DR3)- DQA1*05-DQB1*02 |
| Moderately increased risk | 194 *(44.3)* | homozygosity for either of the two risk haplotypes above or for DRB1*04:01/2/5-DQA1*03-DQB1*03;02 combined with a neutral haplotype |
| Slightly increased risk | 117 (26.7) | (DR3)-DQA1*05-DQB1*02 or DRB1*04:04-DQA1*03-DQB1*0302 with a neutral haplotype or the DRB1*0401/2/5-DQA1*03-DQB1*03:02 / (DR13)- DQB1*0603 genotype |
| Neutral risk | 20 (4.6) | 9 subjects had the combination of DRB1*04:04-DQA1*03-DQB1*03:02 with a protective haplotype: (DR13)-DQB1*06:03 (n=6), (DR14)-DQB1*05:03 (n=1)  (DR7)-DQA1*02:01-DQB1*03:03 (n=2);  6 subjects had a combination of DRB1*04:01-DQA1*03-DQB1*03:02 with a protective haplotype: (DR7)-DQA1*02:01-DQB1*03:03;  4 subjects had a combination of (DR3)-DQA1*05-DQB1*02 with DRB1*04:03-DQA1*03-DQB1*03:02  1 subject had DRB1*04:07-DQA1*03-DQB1*03:02 / (DR8)-DQB1*04 genotype |
| Slightly decreased risk | 8 (1.8) | DRB1*04:03-DQA1*03-DQB1*03:02 with (DR8)-DQB1*04 (n=2), (DR13)-DQB1*06:04 (n=2), (DR9)-DQA1*03-DQB1*03:03 (n=1), (DR1/10)-DQB1*05:01 (n=2), (DR7)-DQA1*02:01-DQB1*02 (n=1) |
| Strongly decreased risk | 1 (0.2) | DRB1*04:03-DQA1*03-DQB1*03:02/(DR13)-DQB1*06:03 |

**Notes:** The groups with a neutral risk, slightly decreased risk and strongly decreased risk were combined, thus resulting in a total of four different risk groups.
